# Letter to editor: Blood pressure, hypertension and lead exposure

**DOI:** 10.1186/s12940-018-0364-3

**Published:** 2018-02-19

**Authors:** Wen-Yi Yang, Jan A. Staessen

**Affiliations:** 1 Studies Coordinating Centre, Research Unit Hypertension and Cardiovascular Epidemiology, KU Leuven Department of Cardiovascular Sciences, University of Leuven, Campus Sint Rafaël, Kapucijnenvoer 35, Box 7001, BE-3000 Leuven, Belgium; 2 Cardiovascular Research Institute Maastricht (CARIM), Maastricht University, Maastricht, The Netherlands

**Keywords:** Ambulatory blood pressure monitoring, Blood pressure, Environmental medicine, Hypertension, Lead

## Abstract

A significant association of office diastolic blood pressure with low-level blood lead exposure was reported in a Brazilian adult population. However, caution should be taken to interpret these results. The multivariable-adjusted association with blood pressure was positive for diastolic blood pressure, but inverse for systolic blood pressure. The association sizes were infinitesimal without clinical relevance. The outcome measures, i.e. blood pressure and the prevalence of hypertension were analysed across categories of the blood lead distribution – not in relation to blood lead as continuous variable. Blood pressure was the average of two oscillometric office readings, whereas ambulatory monitoring is the state-of-the-art.

## Dear Editor

We read with great interest the article on the association of blood pressure with blood lead in a Brazilian urban population sample [1]. The title of Table 3 in this article is ambiguous, because a cross-sectional study cannot detect a change in blood pressure, but only a difference associated with the blood lead level. The outcome variables, i.e. blood pressure and the prevalence of hypertension were evaluated across quartiles of the blood lead distribution or by contrasting people below the 10th and above the 90th of blood lead. Whether analyses relating blood pressure to blood lead as a continuous variable substantiated the categorical results was not reported. The association sizes between blood pressure and blood lead as reported in Table 3 were infinitesimal and inconsistent with an inverse association for systolic blood pressure and a positive association for diastolic blood pressure. We believe that the blood pressure estimates reported in Table 3 express the association for a 1-unit increase in the logarithmically transformed blood lead within each quartile, i.e. a 10-fold increase on the arithmetic scale. Moreover, the authors analysed blood pressure as the logarithmically transformed value of the inverse distribution, i.e. log_10_ (1/blood pressure). This might explain the small effect sizes. Assuming that this is correct, the true association size for diastolic blood pressure in quartile 4 of blood lead is probably 0.85 mmHg (95% confidence interval, 0.81–0.91) instead of 0.07 mmHg (0.04–0.09) for model 1 and 0.87 mmHg (0.81–0.91) instead of 0.06 mmHg (0.04–0.09) for model 2. The title of Table 4 reads “OR (95% CI) of hypertension by blood lead quartiles (μg/dL)”, but Table 4 also includes the odds ratios contrasting the top vs. the lowest tenth of the blood lead distribution. Remarkably, the odds ratios across quartiles of blood lead reveal a curvilinear association showing lower risk of hypertension in the medium-low and medium-high quartiles and higher risk in the top quartile compared with the bottom quartile [1]. This observation again highlights the necessity to analyse blood lead as a continuous exposure variable and to explore whether a linear model is appropriate.

We recently reported on the association of blood pressure with blood lead in 236 newly employed men (mean age, 28.6 years) without previous lead exposure not treated for hypertension [2]. The geometric mean of blood lead was 4.5 μg/dL. In multivariable-adjusted analyses, office systolic blood pressure and the awake and asleep ambulatory blood pressures were not associated with blood lead. For a 2-fold increase in blood lead, the association size of office diastolic blood pressure was 0.87 mmHg (0.03–1.72). The prevalence of the white-coat effect was 19.1%. When corrected for the white-coat effect, the associations between office diastolic blood pressure and blood lead lost significance.

In conclusion, we are looking forward to reading the reply of our Brazilian colleagues on our comments. In addition, we feel that future studies on the association between blood pressure and blood lead should account for out-of-the-office blood pressure, preferentially measured by ambulatory monitoring as recommended in all current guidelines [3–7] for the diagnosis and management of hypertension, including the Brazilian guideline [7].

**Acknowledgements**

The authors acknowledge the expert clerical assistance of Vera De Leebeeck, Yvette Piccart and Renilde Wolfs.

**Funding**

The European Union (HEALTH-F7–305507 HOMAGE) and the European Research Council (Advanced Researcher Grant 2011–294713-EPLORE and Proof-of-Concept Grant 713601-uPROPHET) and the Fonds voor Wetenschappelijk Onderzoek Vlaanderen, Ministry of the Flemish Community, Brussels, Belgium (G.0881.13) currently support the Studies Coordinating Centre in Leuven. ILZRO supports SPHERL study of our group by an unrestricted research grant.

**Availability of data and materials**

Data sharing is not applicable to this article as no datasets were “generated” for this letter. All of the data we referred in the letter are properly cited and already publicly available.

**Authors’ contributions**

YWY and JAS drafted and has approved the final form of this letter.

**Ethics approval and consent to participate**

Not applicable.

**Consent for publication**

Not applicable.

**Competing interests**

The authors declare that they have no competing interests.

## Author’s response to “Letter to the editor: Blood pressure, hypertension and lead exposure”

Ana Carolina Bertin de Almeida Lopes^1^ acalmeida0104@gmail.com, Ellen Kovner Silbergeld^2^ esilber2@jhu.edu, Ana Navas-Acien^2^ an2737@cumc.columbia.edu, Rachel Zamoiski^2^ rachel.zamoiski@gmail.com, Airton da Cunha Martins Junior^3^ airtoncmjr@gmail.com, Alissana Ester Iakmiu Camargo^4^ alissanaester@hotmail.com, Mariana Ragassi Urbano^1,5^ mariana.ragasi@gmail.com, Arthur Eumann Mesas^1,6^ aemesas@gmail.com, Monica Maria Bastos Paoliello^1*,7^ monibas2@gmail.com

^1^Graduate Program in Public Health, Center of Health SciencesState University of LondrinaLondrina, PR, Brazil

^2^Department of Environmental Health Sciences, Johns Hopkins Bloomberg School of Public HealthBaltimore, MD, USA

^3^Graduate Program in ToxicologyFaculty of Pharmaceutical Sciences of University of São PauloRibeirao Preto, SP, Brazil

^4^Graduate Program in Health SciencesState University of LondrinaLondrina, PR, Brazil

^5^Department of StatisticsState University of LondrinaLondrina, PR, Brazil

^6^Department of Public HealthState University of LondrinaLondrina, PR, Brazil

^7^Department of Molecular Pharmacology, Albert Einstein College of MedicineBronx, NY 10461, USA

**Abstract**

**Background**

We would like to thank Drs. Yang and Staessen for their insight on our article entitled “Association between blood lead and blood pressure: a population-based study in Brazilian adults”. We previously reported that low blood lead levels (BLL) were associated with diastolic blood pressure and with the odds for hypertension.

**Comments**

In response, we clarify the epidemiological relevance, as well as the strength and consistency of the findings presented in our original manuscript. In addition, we provide further details on the soundness of the statistical procedures described in the original article.

**Conclusion**

The re-analysis corroborates the original findings and their epidemiological relevance.

**Keywords**

Blood lead, Blood pressure, Hypertension, Population-based

**Background**

In the study entitled “Association between blood lead and blood pressure: a population-based study in Brazilian adults” [1], we evaluated a randomly selected urban population. We found a positive association between blood lead and diastolic blood pressure, and with the odds for hypertension. After considering the comments raised by Drs. Yang and Staessen, we justify the epidemiological relevance, the strength and consistency with our findings, in addition to providing additional details on the soundness of the statistical procedures.

**Comments**

We would like to thank Drs. Yang and Staessen for their comments made in relation to our article on the association of blood pressure with blood lead [1], and we appreciate the opportunity to answer to the issues raised in their letter.

The decision to present the relationship of the outcome variables (blood pressure and prevalence of hypertension) across quartiles of blood lead or by comparing the low with the higher percentiles was undertaken to enable a flexible evaluation of the dose-response relationship based on categories that are properly distributed according to values found in the studied population itself, in order to test the observed differences. This is a common method in environmental epidemiology and is often found in those studies that have included blood lead levels to test for associations between blood lead and blood pressure outcomes [8–10].

Drs. Yang and Staessen’s comments on our statistical analysis seem to be based on the understanding that we used an inverse log scale, which is not the case. We present a detailed description of the analyses we have performed.

For systolic blood pressure, the best transformation to approximate normality was an inverse transformation (i.e. 1/(systolic blood pressure)) and the results are shown in Table 3 of the original manuscript. For systolic blood pressure the residuals with a log-transformation would statistically significant different from normal (*p*-value with the Shapiro-Wilk test < 0.001), while with the inverse transformation the residuals would no longer be different from the normal distribution (*p*-value 0.822). For diastolic blood pressure the best transformation to approximate normality was a log transformation (*p*-value = 0.637 with the Shapiro-Wilk test).

The mean difference for inverse blood pressure levels across blood lead quartiles is 0.0000 (reference), − 0.0002034, − 0.0002281 and − 0.0003540 for lead quartiles 1, 2, 3 and 4, respectively. In the original scale, systolic blood pressure levels varied between 91 and 231 mmHg. After the inverse transformation, the transformed data varied between 0.010989 (1/91) and 0.004329 (1/231), implying that lower values in the transformed scale are equivalent to higher values in the original scale. For the parameters estimates of the blood lead quartiles, the lowest is for quartile 4, “-0.0003540”, implying that higher levels of lead are associated with higher systolic blood pressure.

The mean difference in diastolic blood pressure in log-units was 0.000 (reference), 0.0390886, 0.0383756, and 0.071560 for lead quartiles 1, 2, 3 and 4, respectively. In the original scale, diastolic blood pressure varied between 51.5 and 125.5 mmHg. Using the log transformation, the transformed data varied between 3.49 (log (51.5)) and 4.83 (log (125.5)). For the parameters estimates of the blood lead quartiles, the higher parameter is for quartile 4, “0.0715607”, implying that higher levels of blood lead are associated with higher diastolic blood pressure. For an easier interpretation we recommend to exponentiate the coefficients, which for quartile 4 would correspond to 1.074. This effect estimate now represents a ratio of the geometric mean comparing quartile 4 to 1, and can also be interpreted as diastolic blood pressure levels being 7.4% higher for participants in the 4 to 1 quartile of blood lead levels. All analyses were carefully redone and confirmed.

With regard to the comment on the size of the results observed in the multivariable adjusted analysis, we believe that the difference in diastolic blood pressure found in individuals with higher blood lead levels is not unexpected in populations with the blood lead levels found in this population. We noted that the magnitude of the results we found is small and is below the levels of blood pressure that have clinical relevance. However, from a population perspective, we consider that a shift in the distribution of a continuous variable, such as blood pressure, can in fact result in increased risks of chronic diseases, including cardiovascular outcomes among those persons whose blood pressures are at the higher end of the scale. This has been demonstrated for the effects of lead on children’s neurocognitive performance and in studies of lead and blood pressure and lead and stroke [8, 11, 12]. We understand that knowledge about all factors potentially related to the increase of the frequency of certain diseases, even those with small effects, is of great importance. This applies specifically to diseases such as hypertension, for which the etiology can include exposures to multiple risk factors that may act independently or in an additive or synergistic manner.

Regarding the title attributed to Table 3, we agree that it is more appropriate to characterize it as “difference” rather than “change”.

Regarding the blood pressure measurement, we are aware that ambulatory monitoring provides a better assessment on blood pressure levels, including the reduction of the white coat effect [13]. In our study, blood pressure measurements were taken in accordance with the VI Brazilian Guidelines on Hypertension, with a proper equipment (Omron HEM 742), and at least three measurements were obtained [14]. Furthermore, the classification of hypertension followed the same cutoff values extant at the time (systolic blood pressure of 140 mmHg or higher, diastolic blood pressure of 90 mmHg or higher, or use of antihypertensive medication), used in most of the studies broadly published in the literature [9, 15–17].

We agree with the importance of the results recently reported by Yang et al., in which the correction for the “white-coat effect” resulted in loss of statistical significance in the association between blood lead and office diastolic blood pressure [2]. We did not consider this the “white-coat effect” in our analysis. Our study included several adjustment variables, related to sociodemographic, clinic and health characteristics, obtained from a census based randomized selection of Brazilian adults.

**Conclusion**

Taken together, we believe in the strength and consistency of our study, despite the relatively small association size. Furthermore, our findings are consistent with multiple epidemiologic and experimental studies suggesting that exposure to lead is causally related to elevated blood pressure levels [15]. Thus, we trust that the present results may be compared with those reported in similar studies with environmentally exposed populations.

## References

[1] de Almeida Lopes ACB, Silbergeld EK, Navas-Acien A, Zamoiski R, Martins ADCJ, Camargo AEI, Urbano MR, Mesas AE, Paoliello MMB (2017). Association between blood lead and blood pressure: a population-based study in Brazilian adults. Environ Health.

[2] Yang WY, Efremov L, Mujaj B, Zhang ZY, Wei FF, Huang QF, Thijs L, Vanssche T, Nawrot TS, Staessen JA (2018). Association of office and ambulatory blood pressure with blood lead in workers prior to occupational exposure. J Am Soc Hypertens.

[3] Siu AL (2015). On behalf of the U.S.Preventive services task force. Screening for high blood pressure in adults: U.S. preventive services task force recommendation statement. Ann Intern Med.

[4] Leung AA, Nerenberg K, Daskalopoulou SS, McBrien K, Zarnke KB, Dasgupta K, Cloutier L, Gelfer M, Lamarre-Cliche M, Milot A, Bolli P, Tremblay G, McLean D, Tobe SW, Ruzicka M, Burns KD, Vallée M, Prasad GVR, Lebel M, Feldman RD, Selby P, Pipe A, Schiffrin EL, McFarlane PA, Oh P, Hegele RA, Khara M, Wilson TW, Penner SB, Burgess E, Herman RJ, Bacon SL, Rabkin SW, Gilbert RE, Campbell TS, Grover S, Honos G, Lindsay P, Hill MD, Coutts SB, Gubitz G, Campbell NRC, Moe GW, Howlett JG, Boulanger JM, Prebtani A, Larochelle P, Leiter LA, Jones C, Ogilvie RI, Woo V, Kaczorowski J, Trudeau L, Petrella RJ, Hiremath S, Drouin D, Lavoie KL, Hamet P, Fodor G, Grégoire JC, Lewanczuk R, Dresser GK, Sharma M, Reid D, Lear SA, Moullec G, Gupta M, Magee LA, Logan AG, Harris KC, Dionne J, Fournier A, Benoit G, Feber J, Poirier L, Padwal RS, Rabi DM, for the CHEP Guidelines Task Force (2016). Hypertension Canada's 2016 Canadian hypertension education program guidelines for blood pressure measurement, diagnosis, assessment of risk, prevention, and treatment of hypertension. Can J Cardiol.

[5] National Institute for Health and Clinical Excellence (NICE) (2011). The clinical management of primary hypertension in adults.

[6] O'Brien E, Parati G, Stergiou G, Asmar R, Beilin L, Bilo G, Clement D, de la Sierra A, de Leeuw P, Dolan E, Fagard R, Graves J, Head G, Imai Y, Kario K, Lurbe E, Mallion JM, Mancia G, Mengden T, Myers M, Ogedegbe G, Ohkubo T, Omboni S, Palatini P, Redon J, Ruilope L, Shennan A, Staessen JA, van Montfrans G, Verdecchia P, Waeber B, Wang J, Zanchetti A, Zhang Y, on behalf of the European Society of Hypertension Working Group on Blood Pressure Monitoring (2013). European Society of Hypertension position paper on ambulatory blood pressure monitoring. J Hypertens.

[7] Malachias MVB, Gomes MAM, Nobre F, Alessi A, Feitosa AD, Coelho EB (2016). 7th Brazillian guideline of arterial hypertension: chapter 2 - diagnosis and classification. Arq Bras Cardiol.

[8] Nash D, Magder L, Lustberg M, Sherwin RW, Rubin RJ, Kaufmann RB (2003). Blood lead, blood pressure, and hypertension in perimenopausal and postmenopausal women. JAMA.

[9] Lee B-K, Ahn J, Kim N-S, Lee CB, Park J, Kim Y (2016). Association of Blood Pressure with exposure to lead and cadmium: analysis of data from the 2008–2013 Korean National Health and nutrition examination survey. Biol Trace Elem Res.

[10] Scinicariello F, Yesupriya A, Chang MH, Fowler BA (2010). Modification by ALAD of the association between blood lead and blood pressure in the U.S. population: results from the third National Health and nutrition examination survey. Environ Health Perspect.

[11] Lustberg M, Silbergeld E (2002). Blood lead levels and mortality. Arch Intern Med.

[12] Pirkle JL, Schwartz J, Landis JR, Harlan WR (1985). The relationship between blood lead levels and blood pressure and its cardiovascular risk implications. Am J Epidemiol.

[13] Grossman E (2013). Ambulatory blood pressure monitoring in the diagnosis and Management of Hypertension. Diabetes Care.

[14] SBH. Sociedade Brasileira de Hipertensão. VI Diretrizes BrasileIiras de Hipertensão. Revista Hipertensão. 2010;13(1)

[15] Navas-Acien A, Guallar E, Silbergeld EK, Rothenberg SJ (2007). Lead exposure and cardiovascular disease--a systematic review. Environ Health Perspect.

[16] Hara A, Thijs L, Asayama K, Gu YM, Jacobs L, Zhang ZY (2015). Blood pressure in relation to environmental lead exposure in the national health and nutrition examination survey 2003 to 2010. Hypertension.

[17] Bushnik T, Levallois P, D'Amour M, Anderson TJ, McAlister FA (2014). Association between blood lead and blood pressure: results from the Canadian health measures survey (2007 to 2011). Health Rep.

